# Effects of the *DUSP6* gene on the proliferation and differentiation of porcine subcutaneous preadipocytes

**DOI:** 10.5713/ab.25.0175

**Published:** 2025-06-24

**Authors:** Xiangxiang Yang, Xiaohan Sun, Zimeng Du, Jundong Yi, Qiuyan Wang, Ding Yin, Yalan Zhang, Xiaoling Ding, Xianrui Zheng, Xiaodong Zhang, Zongjun Yin, Yueyun Ding

**Affiliations:** 1 College of Animal Science and Technology, Anhui Agricultural University, Hefei, China; 2 Anhui Province Key Laboratory of Local Livestock and Poultry Genetic Resource Conservation and Bio-Breeding, Anhui Agricultural University, Hefei, China

**Keywords:** Cell Proliferation and Differentiation, *DUSP6*, Pig, Preadipocytes

## Abstract

**Objective:**

Dual-specificity protein phosphatase 6 (*DUSP6*), also known as mitogen-activated protein kinase phosphatase 3 (*MKP-3*), was considered as a functional candidate gene for white fat accumulation in mice. However, the physiological function of the *DUSP6* gene on white adipocyte adipogenesis in farm animals remains unknown. In this study, we aimed to clarify the effect of *DUSP6* on porcine subcutaneous preadipocyte proliferation and differentiation.

**Methods:**

We first make clear that the patterns of *DUSP6* expression is associated with fat contents in porcine fat deposition related tissues. Porcine subcutaneous preadipocytes were isolated and induced to differentiation. Small interfering RNAs were applied to deplete *DUSP6*. MTT assay, CCK-8 analysis, Oil Red O staining, triglyceride determination and reverse transcription quantitative polymerase chain reaction were applied to study the regulatory role of *DUSP6* during adipocyte adipogenesis in pigs.

**Results:**

We found that the expression levels of *DUSP6* were significantly higher in backfat and *longissimus dorsi* tissues from fat-type pigs than in those from lean-type pigs. Consistently, the significantly induced expression of *DUSP6* was also observed in differentiated adipocytes. In addition, knockdown of *DUSP6* greatly inhibited preadipocytes proliferation, through the decreased cell viability and downregulated mRNA expressions of cell proliferation-associated genes, including *PCNA*, *CDK1*, *CDK2*. Furthermore, knockdown of *DUSP6* significantly inhibited preadipocytes differentiation, as evidenced by markedly reduced lipid droplet formation, attenuated triglyceride accumulation and downregulated expression levels of adipogenic transcription masters (*PPARγ*, *C/EBPβ*, *FASN* and *FABP4*) in *DUSP6* knockdown cells.

**Conclusion:**

Our results demonstrate that *DUSP6* is required for white adipocyte adipogenesis in pigs.

## INTRODUCTION

Over the past few decades, obesity has become a significant global health challenge with a rapidly rising incidence. Obesity represents a complex, heterogeneous, chronic, and progressive disease [1], leading to severe conditions such as hypertension, hyperlipidemia, Type 2 diabetes, stroke, metabolic syndrome, asthma, and cancer [2], which contribute to an increase in morbidity and mortality. Urgent and sustained attention of obesity-related health risks are most warranted [3]. As the burden of obesity is increasing steadily, its direct and indirect costs to society are substantially immense [4].

Obesity is thought to be due to the excessive accumulation of white adipose tissue [5]. Adipose tissue expansion and development occur through increasing in the number of preadipocytes, as well as differentiation of preadipocytes into mature adipocytes capable of triglyceride (TG) storage, metabolism, production of adipokines, which is known as adipogenesis [6]. Thus, the proliferation and differentiation of preadipocytes in white fat tissue are contributed to adipose tissue development and even body weight gain. In addition, adipose tissue deposition is highly correlated with meat quality and production efficiency in farm animals [7–9]. Pigs are the most important meat livestock worldwide. Fat deposition-related traits, including backfat thickness, carcass leanness, feed efficiency, and meat quality, are of economic importance in swine breeding [10,11]. Meanwhile, pigs are not only similar to humans in terms of the anatomy and physiology of the cardiovascular, but also exhibit a high degree of similarity in the process of lipid metabolism, that is widely used to investigate the mechanism of fat deposition and obesity [12]. Therefore, the exploration mechanism of porcine preadipocyte proliferation and differentiation has significant implications for human obesity-related diseases and pig breeding production.

The process of preadipocytes proliferation and differentiation is a change in gene expression patterns. Intensive efforts have been made to reveal the genetic basis of adipogenesis, especially in terms of differentiation, and several central transcription factors (TFs) including peroxisome proliferator-activated receptor γ (*PPARγ*), CCAAT/enhancer-binding protein (C/EBP) α, β, δ, and lipogenic-related genes including fatty acid binding protein 4 (*FABP4*) and fatty acid synthase (*FASN*), have been characterized as the master adipogenic markers [13,14]. However, adipogenesis is a tightly orchestrated cellular differentiation process regulated by a cascade of TFs; the regulatory network of adipogenesis is far from complete. Alternatively, other studies have also shown that many regulatory genes, such as *PABPN1* [15], *E2F5* [16], and *ADAR1* [17], can regulate adipocyte development. Thus, identification of the involved genes and networks, as well as exploration of the genetic regulatory mechanisms that control porcine adipogenesis, is required to better understand fat deposition in pigs.

Dual-specificity protein phosphatase 6 (*DUSP6*), also known as mitogen-activated protein kinase phosphatase-3 (*MKP-3*), is a member of the dual-specificity protein phosphatases (DUSPs) family [18]. By interacting with multiple nodes of Notch1 [19], CYP4A [20], MAPK and PI3K/AKT signaling pathways [21], *DUSP6* is involved in modulating diverse cellular processes, such as cell growth, proliferation, differentiation, migration, invasion, metabolism (lipid accumulation in HepG2 cells) [19–22]. It is also associated with the pathological processes of obesity [23], nonalcoholic fatty liver [20], cognitive impairment [21], and multiple types of cancers [22]. Unlike its dual manner functions, pro-oncogenic or tumor-suppressive, in the pathological processes of cancers, the positive function of *DUSP6* in white adipocyte adipogenesis is reported. Mice lacking *DUSP6/8* have enhanced resistance to diet-induced obesity, with the dramatically reduced serum TG, lipid content in the liver and visceral adipose tissues [24]. *DUSP6* mRNA level has been previously reported to increase in white adipose tissue of ob/ob and db/db mice [25]. The level of *DUSP6* protein expression significantly increases at the early stage of 3T3-L1 adipogenesis (2 days after induction); *DUSP6* deficiency is further linked with impaired *in vitro* adipocyte differentiation in a subline of 3T3-L1 and in the isolated preadipocytes (the stromal vascular cells isolated from gonadal adipose tissue of *Dusp6−/−*mice) [26]. However, the molecular mechanism of *DUSP6* deficiency in inhibiting white adipocyte differentiation is not fully understood. Furthermore, little is known on the actual role of *DUSP6* in white preadipocyte adipogenesis in farm animals, and further study is warranted.

It is thought that *DUSP6* may play a crucial role in white adipose accumulation. Herein, the aims of this study were to: (1) identity the growth curve and differentiation ability of primary subcutaneous preadipocytes cultured from newborn Huoshou black piglets; (2) detect the expression patterns of the *DUSP6* gene in the fat deposition related tissues from fat-type and lean-type pigs, and in differentiating preadipocytes; (3) examine the effects on the proliferation and differentiation of preadipocytes after knockdown of the *DUSP6* gene. In this study, we initially investigated the *DUSP6* gene effect on the porcine adipocyte adipogenesis to provide scientific clues for the improvement of pig meat quality and the treatment of obesity-related diseases.

## MATERIALS AND METHODS

### Sample collection for reverse transcription quantitative polymerase chain reaction analysis

Our research was approved by the Anhui Agricultural University Animal Ethics Committee under permission No. KKLL2025014. Backfat and *longissimus dorsi* tissues of 5 Youhulu type Huoshou Black (fat type) and 5 Landrace finishing female pigs (lean type) with similar body weights (113.00± 7.24 kg, n = 10), with extremely divergent back fat thickness and IMF (45.36 mm±3.80 *vs.* 26.00 mm±1.63, n = 5, p<0.01; 8.54%±0.31 *vs.* 2.53 %±0.14, n = 5, p<0.01), were collected and kept in our lab at −80°C until further use for reverse transcription quantitative polymerase chain reaction (RT-qPCR) analysis.

### Cell isolation, culture, induction differentiation, and transfection

Preadipocytes were isolated from 3-day-old Huoshou black piglets as follows. Under sterile conditions, subcutaneous adipose tissues from the back and neck of piglets were collected and washed three times using PBS containing 5% penicillin/streptomycin (Gibco). The adipose tissue samples were then sheared into pieces, and were sufficiently digested with collagenase Type I (Solarbio) at 37°C for 1 h. After digestion, the mixture was filtered to remove the undigested fractions, and then centrifuged at 1,500 r/min for 10 min to obtain the preadipocytes. The preadipocytes were then resuspended and seeded in the complete medium comprising 89% DMEM/F12 (Gibco), 10% FBS (Gibco) and 1% penicillin/streptomycin. The cells were then incubated at 37°C in a cell culture incubator using 5% CO_2_. Culture medium was changed every 2 days.

To induce differentiation, 2 days after confluence, cells were supplied with differentiation medium (DMEM/F12 containing 10 % FBS plus 5 μg/mL insulin [Solarbio], 1 μM dexamethasone [Solarbio], 0.5 mM IBMX [Solarbio]) for 2 days, and then cultured in maintain medium (DMEM/F12 containing 10 % FBS plus 5 μg/mL insulin) for another 6 days, and medium was replaced every 2 days. The adipogenic differentiation spanned around 8 days.

For *DUSP6* knockdown, cells at 60% confluence were transfected by small-interfering RNAs (siRNAs) or negative control (NC) using the Lipofectamine 2000 transfection kit (Invitrogen) according to the manufacturer’s instructions. The siRNAs used in this study were designed by GenePharma and contained three *DUSP6* gene siRNA sequences and an siRNA NC (Table 1).

### MTT assays measure the growth curve of primary cultured preadipocytes

Pig subcutaneous preadipocyte proliferative capacity was examined by performing MTT assays on 1, 2, 3, 5, 7, 9, 11, 13 days after cell attachment. Cells were seeded into 96-well plates at a density of 5,000 cells/well. Firstly, 10 μL of prepared 5mg/mL MTT solution was added and incubated at 37°C and 5% CO_2_ for 4 h. Then, MTT solution was discarded and 100 μL of DMSO was added to the wells for mixing. Finally, optical density values were subsequently measured at 490 nm using a microplate reader (Thermo Fisher Scientific).

### CCK-8 assays examine the effect of *DUSP6* on preadipocyte proliferation

Briefly, preadipocytes were seeded in 96-well plates and transfected with siRNA or NC at 60% cell density. Six hours later, the medium was changed to the complete medium. After incubation for 48 h, 10 μL of CCK reagent (Beyotime) was added to each well and incubated at 37°C and 5% CO_2_ for 2 h. Then the absorbance at 450 nm were measured by the microplate reader.

### Oil Red O staining and triglyceride determination

At the 7th day postinduction, the siRNA or NC treated mature adipocytes were stained using an Oil Red O kit (Solarbio) according to the manufacturer’s instructions, and morphologically viewed under a fluorescence microscope (Zeiss Axio Vert A1). To quantify the lipid droplets, cellular Oil Red O was extracted using 100% isopropanol and analyzed with optical absorbance at 510 nm.

For TG determination, the siRNA or NC treated mature adipocytes were collected at the 7th day postinduction and were lysed with 2% Trition. Cell TG content was determined using a Triglyceride kit (JianCheng) following the manufacturer’s procedure.

### RNA extraction and reverse transcription quantitative polymerase chain reaction

Total RNA was extracted using TRIzol Reagent (Biomed), and was reversed into cDNA using the reverse transcription kit (Xinbei) after RNA integrity and RNA concentration analysis. RT-qPCR was performed using the SYBR Green PCR Master Mix (Xinbei) by using a CFX96 Touch Real-Time PCR Detection System (Bio-Rad). RT-qPCR primer information was listed in Table 2. Target gene expression levels were analyzed by the 2^−ΔΔCt^ method using *GAPDH* to normalize expression.

### Statistical analysis

GraphPad Prism (Version 9; GraphPad Software) was used for statistical analysis. Statistical comparisons between two groups were made using the unpaired 2-tailed Student’s t test, multiple comparisons were performed using one-way ANOVA followed by Dunnett's or Tukey's multiple comparison test as recommended by GraphPad Prism. In all cases, levels of statistical significance were set at * p<0.05, ** p<0.01, and *** p<0.001. All data are presented as the mean±standard deviation. All experiments were performed (at least) in triplicate.

## RESULTS

### Validation of adipogenic differentiation ability of porcine subcutaneous preadipocytes

**Figure 1:**
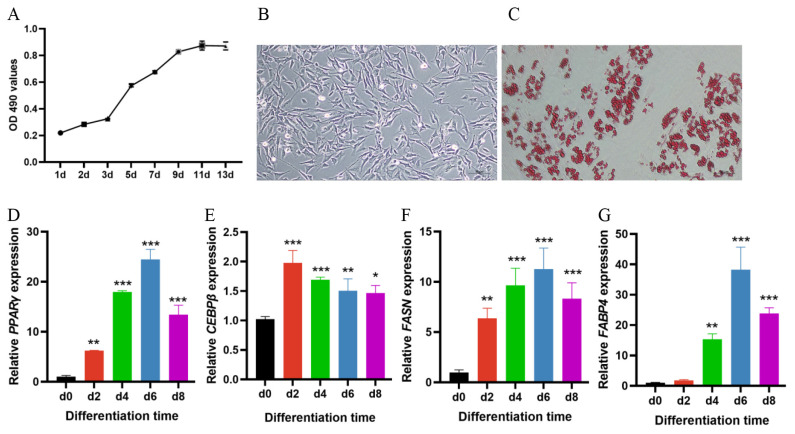
Validation of adipogenic differentiation ability of the porcine subcutaneous preadipocytes. (A) Growth curve of primary cultured porcine preadipocytes. (B) Cell morphology of undifferentiated preadipocytes, bar = 50 μm. (C) Oil red O staining of lipid droplets on the 8th day of differentiation, bar = 50 μm. (D–G) The relative expression levels of *PPARγ*, *C/EBPβ*, *FASN* and *FABP4* in porcine preadipocytes after inducing differentiation on 0, 2nd, 4th, 6th, 8th day (Significance vs. 0 day, * p<0.05, ** p<0.01, and *** p<0.001).

In order to identify the growth of primary cultured porcine subcutaneous preadipocytes and whether they can stably differentiate into mature adipocytes, we measured the growth curve, observed cell morphology and images of ORO staining, and also detected the expression trends of adipogenic marker genes during the differentiation. The growth curve showed an “S” shape (Figure 1A). Observation and analysis of cell morphology revealed that porcine preadipocytes had a homogeneous irregular spindle morphology when they were undifferentiated (Figure 1B). Successful differentiation was proved by positive ORO staining (Figure 1C), and significantly increases in the preadipocyte differentiation key genes expression, with the highest *C/EBPβ* level on day 2, and the highest *PPARγ, FASN* and *FABP4* levels on day 6 (Figures 1D–1G). Therefore, we conclude that the cells had a high ability of adipogenic differentiation and can be used for subsequent functional studies.

### Expression profile of *DUSP6* in fat deposition related tissues and cells

**Figure 2:**
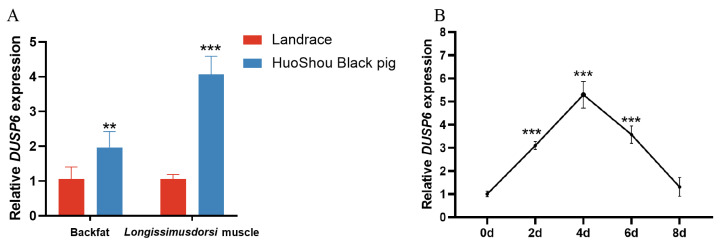
Expression profile of *DUSP6* in tissues and cells. (A) *DUSP6* was highly expressed in backfat and *longissimus dorsi* tissues of fat-type pigs (Significance vs. Landrace, ** p<0.01, and *** p<0.001). (B) *DUSP6* expression in porcine preadipocyte after inducing differentiation on 0, 2nd, 4th, 6th, 8th day (Significance vs. 0 day, *** p<0.001).

To explore the expression characteristics of *DUSP6*, we first investigated *DUSP6* expression levels in backfat and *longissimus dorsi* tissues from Youhulu type Huoshou Black pigs, a fat-type Chinese native pig breed and Landrace, a well-known commercial lean-type pig. We found that the expression of *DUSP6* were dramatically higher in backfat and *longissimus dorsi* tissues from Youhulu type Huoshou Black pigs than in those from Landrace pigs (Figure 2A).

Next, we investigated *DUSP6* expression levels every two days during adipocyte differentiation. In the adipocyte differentiation model, *DUSP6* expression showed significant variation and reached the highest level on day 4 of adipogenic differentiation (Figure 2B), revealing a possible positive role of *DUSP6* in porcine adipogenesis.

### *DUSP6* interference inhibits porcine subcutaneous preadipocyte proliferation

**Figure 3:**
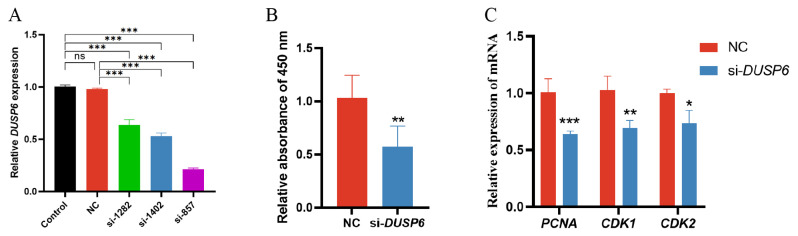
*DUSP6* promotes porcine subcutaneous preadipocyte proliferation. (A) si-857 is the best interfering sequence for *DUSP6* (Significance vs. Control/NC, ns, p>0.05; *** p<0.001). (B) The cell viability of preadipocyte after transfection for 48 h (Significance vs. NC, ** p<0.01). (C) RT-qPCR detection of mRNA levels of proliferation-related marker genes in adipocytes after transfection for 48 h (Significance vs. NC, * p<0.05, ** p<0.01, and *** p<0.001). NC, negative control; RT-qPCR, reverse transcription quantitative polymerase chain reaction.

With the aim of elucidating the exactly functional roles of *DUSP6* in porcine subcutaneous preadipocyte proliferation, three siRNAs specific for the porcine *DUSP6* gene were designed and used to transfect porcine subcutaneous preadipocytes. The knockdown efficiency of three siRNAs was detected by RT-qPCR analysis at 48 h post-transfection in preadipocytes. The results indicated that si-*DUSP6*-857 had the strongest interference efficiency, with 79% and 78% compared to control and NC, respectively (Figure 3A), and this siRNA was selected as the best candidate siRNA for subsequent studies and was named as si-*DUSP6*.

The CCK-8 assay was used to analyze the proliferative effects of *DUSP6* on porcine subcutaneous preadipocytes. Following *DUSP6* knockdown, cell viability significantly decreased compared to the NC group after 48 h of treatment, indicating that *DUSP6* knockdown inhibited adipocyte proliferation (Figure 3B). Coincidentally, the mRNA levels of important markers of cell proliferation like *PCNA*, *CDK1*, *CDK2* were significantly downregulated due to the knockdown of *DUSP6* in porcine subcutaneous preadipocytes (Figure 3C). Collectively, these data suggested that *DUSP6* is required for porcine subcutaneous preadipocyte proliferation.

### *DUSP6* interference inhibits porcine subcutaneous preadipocyte differentiation

**Figure 4:**
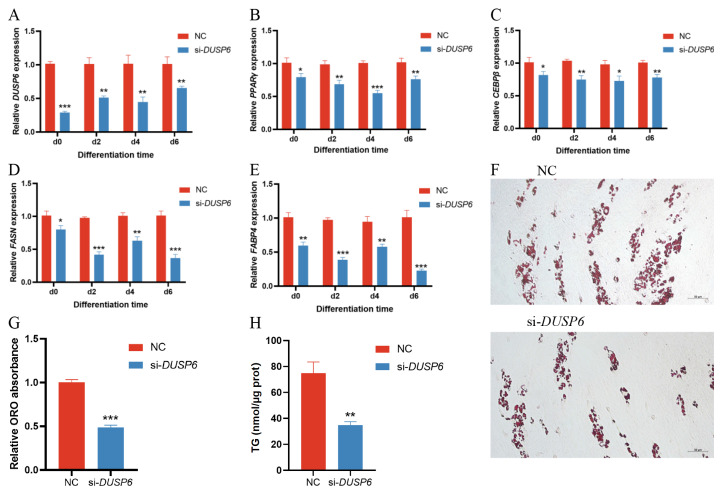
Interference of *DUSP6* inhibits preadipocyte differentiation. (A–E) *DUSP6* and the adipogenic markers (*PPARγ*, *C/EBPβ*, *FASN* and *FABP4*) mRNA levels in adipocytes infected with si-*DUSP6*, NC on day 0 (D0), day 2 (D2), day 4 (D4) and day 6 (D6) after differentiation (Significance vs. NC, * p<0.05, ** p<0.01, and *** p<0.001). (F–H) Oil Red O staining (bar = 50 μm), Oil Red O relative absorbance, and triglyceride content in adipocytes infected with si-DUSP6, NC on the 7th day after differentiation (Significance vs. NC, ** p<0.01, and *** p<0.001). NC, negative control; TG, triglyceride.

Then, to assess the effect of *DUSP6* knockdown on adipocyte differentiation, porcine preadipocytes were transfected with si-*DUSP6*, and induced to differentiate. The expression of *DUSP6* and the differentiation-related biomarkers in different periods of preadipocyte differentiation (days 0, 2,4, 6) were detected by RT-qPCR, and the TG content and lipid droplets were measured on day 7. As demonstrated, si-*DUSP6* significantly decreased the expression of *DUSP6* during adipocyte differentiation (Figure 4A). The levels of adipogenic marker genes *PPARγ*, *C/EBPβ*, *FASN* and *FABP4* during adipocyte differentiation were significantly reduced at the mRNA level following knockdown of *DUSP6*, respectively (Figures 4B–4E). The results of Oil red O staining (Figure 4F), Oil Red O extraction (Figure 4G) and TG assay (Figure 4H) revealed that knockdown of *DUSP6* significantly reduced the number of lipid droplets, the intracellular lipid content and TG accumulation in porcine adipocytes. Collectively, these data suggested that *DUSP6* knockdown impaired the differentiation of porcine preadipocytes.

## DISCUSSION

*DUSP6* plays critical roles in development and disease, including an increasing connection to cancer progression [19,27,28]. Literature has also confirmed that *DUSP6* is involved in white fat deposition in mice. Obesity in mice increased the *DUSP6* (*MKP-3*) protein content in the hypothalamus, this hypothalamic upregulation led to an increase of food intake, adiposity, and body weight [29]. *MKP-3* deficient mice are protected from several side effects of chronic Dex exposure, such as body weight gain, adipose tissue enlargement, hepatic lipid accumulation, and insulin resistance [30]. Mice lacking *DUSP6/8* were resistant to high-fat diet-induced obesity [24]. The absence of *MKP-3* also reduces adiposity in mice, possibly by repressing adipocyte differentiation [26]. However, the functional role of *DUSP6* in white adipocyte adipogenesis in farm animals remains unknown. Here, we demonstrate for the first time that *DUSP6* regulates the proliferation and differentiation of porcine subcutaneous preadipocytes. To the best of our knowledge, this is the first report on the role of porcine *DUSP6* in fat formation. The results will facilitate our understanding of obesity in mammals and on the meat quality in domestic animals.

In this study, we first cultured subcutaneous primary preadipocytes from 3-day-old Huoshou black piglets as experimental materials, and then applied exogenous drugs DEX, IBMX, and insulin to induce preadipocytes differentiation. Here, in all tested *in vitro* cell samples, RT-qPCR results revealed that *PPARγ*, *C/EBPβ*, *FASN* and *FABP4* expression were detected and upregulated in the differentiation process, implying that the differentiation model was applicable. Adipogenesis is a multi-step process, including the early (2 days after induction), intermediate (about the 3rd to 5th day after induction), and late stages (approximately after the fifth day of induction) of differentiation [31], which encompasses cascades of TFs for crucial proteins that induce gene expression to form mature adipocytes [32]. In mammalian white adipocytes, *C/EBPβ*, *PPARγ* are considered the key early regulators of adipogenesis, while *FABP4* and *FASN* are responsible for the lipid formation in late stage of differentiation [33]. At the early stage of differentiation, there is a high expression of *C/EBPβ* and *C/EBPδ* in response to hormonal induction, resulting in the induction of PPAR*γ* expression [34]. Upon activation, *PPARγ* induces the expression of many adipocyte genes, including *C/EBPα*, which is considered as a marker of mid stage differentiation [35,36]. The presence of *C/EBPα* may allow continued expression of *PPARγ* and perpetuation of the differentiated state [34]. The newly formed mature adipocytes still maintain a high expression of *PPARγ* and *C/EBPα* [36]. FASN, a key enzyme in the *de novo* synthesis of fatty acids, is involved in white adipocyte adipogenesis as a late adipogenic marker [33]. *FABP4*, also referred to as *AP2*, is expressed late during adipocyte differentiation and has been postulated to play important roles in fatty acid uptake and accumulation [37]. In our results of establishment of porcine subcutaneous preadipocyte differentiation model, the expression of early key differentiation related markers of *C/EBPβ* (significantly upregulated on the 2nd day and with the highest levels on day 2) and *PPARγ* (significantly upregulated on the 2nd day and remained at a relatively high level thereafter), and the expression of the lipogenic-related *FABP4* (significantly upregulated during the 4th to 8th day of differentiation), *FASN* (significantly upregulated on the 2nd day and remained at a relatively high level thereafter) genes were almost consistent with their expression trends in previous white preadipocyte differentiation models. Besides, we also observed undifferentiated cell morphology and the lipid drops by Oil Red O staining assay, combined with results from the *PPARγ*, *C/EBPβ*, *FASN* and *FABP4* RT-qPCR trial, indicating that the cultured preadipocytes had a highly homogeneous morphology and a high ability of adipogenic differentiation.

Gene expression is the basis for function. In the present study, our analyses demonstrated that the expression of *DUSP6* were dramatically higher in backfat and *longissimus dorsi* tissues from Youhulu type Huoshou Black pigs (a fat-type Chinese native pig breed) than in those from Landrace pigs. These results are consistent with previous reports from mouse studies indicating that mice with diet-induced obesity has markedly increased *DUSP6* expression levels in white adipose tissues [25,26,38]. Moreover, our results also revealed that *DUSP6* expression levels were markedly different during the 2nd and 6th day of differentiation, with the sharpest increase on the 4th day, hypothesizing that *DUSP6* may mainly be involved in mid stages of the porcine preadipocytes differentiation process. These data suggest a potential positive role of *DUSP6* in the fat accumulation of pigs.

The proliferation and differentiation of preadipocytes were vital for fat tissue formation, and the rate and number of preadipocyte proliferation determined the degree of adipocytes development [39]. As our data showed, down-regulation of *DUSP6* by *siRNA* resulted in a significant decrease of cell viability and the expression levels of cell cycle-related genes including *PCNA*, *CDK1*, *CDK2*, which are the key components of cell cycle signaling pathway and act as positive regulators in promoting cell cycle progression. PCNA, primarily synthesized during the S phase of the cell cycle, participates in DNA replication and regulates the cell cycle progression [40,41]. CDK1 binds to Cyclin B to form a complex, thereby acquiring kinase activity, which drives the G2/M transition [42]. CDK2 binds to Cyclin E and inactivates the retinoblastoma protein via phosphorylation, thereby initiating the G1/S transition [43]. This is a particularly encouraging result and indicates that *DUSP6* positively regulates porcine preadipocyte proliferation through *PCNA*, *CDK1* and *CDK2*. Similar observations regarding the influence of *DUSP6* on cell proliferation have been documented. Targeted knockdown of *DUSP6* by siRNA significantly inhibited the proliferation of human TPC1 thyroid cancer cells [44]. Knockdown *DUSP6* with siRNA inhibited proliferation, migration, and invasion in gastric cancer cells [45]. Song et al [46] showed that down regulation of *DUSP6* in MDA-MB-231 cells suppressed the cell proliferation, and meanwhile the cells were arrested at G0/G1 phase. This is the first report on the role of *DUSP6* in regulating the growth rate of preadipocytes in farm animals, while, the cellular mechanisms underlying this effect are still need to be further study. These observations suggested the necessity for additional research on how si*DUSP6* affected the cell cycle progression and the expressions of *PCNA*, *CDK1* and *CDK2* in the porcine subcutaneous preadipocyte proliferation process.

In the present study, we demonstrated for the first time that si-*DUSP6*, inhibits adipogenesis in porcine subcutaneous preadipocytes, potentially through hindering the expression of adipogenic marker genes (including *PPAR*γ, *C/EBPβ*, *FASN* and *FABP4*). As our data showed, with the decrease in *DUSP6* during adipocytes differentiation, *PPAR*γ, *C/EBPβ*, *FASN* and *FABP4* genes expression constantly decreased; consequently, the accumulation of the TG content and lipid droplets significantly reduced. This indicated that *DUSP6* functions as a positive regulator in porcine subcutaneous preadipocyte differentiation and lipid accumulation. Our findings are consistent with the observations in 3T3-L1 cells and the stromal vascular cells isolated from gonadal adipose tissue of Dusp6−/− mice, with the white preadipocyte differentiation efficiency compromised by *DUSP6* knockdown [26]. The mechanism by which *DUSP6* regulates white adipocyte adipogenesis has not yet been extensively elucidated at the cellular level. However, it is currently understood that in gonadal and mesenteric fat tissues of MKP-3^−/−^ male mice fed an HFD, *DUSP6*-mediated regulation of adipogenesis operates independently of the ERK pathway [26]. Interestingly, a previous study revealed that *DUSP6* inhibits brown adipocyte differentiation in mice via regulation of ERK phosphorylation [47]. It cannot be excluded that these discrepancies resulted from the different type of cells. In particular, *DUSP6* expression was significantly downregulated during brown adipocyte differentiation compared with white adipogenesis [47]. This could be because gene regulation networks are very complicated. In cells, the expression of genes is regulated by multiple genes and signaling pathways. Therefore, we guessed that the different roles of *DUSP6* in white and brown adipocyte adipogenesis could possibly be due to the complexity of cellular signaling regulatory networks.

Our study had several limitations. It needs to be pointed out that our experiments were performed only *in vitro*. Therefore, it remains to be investigated whether *DUSP6* modulates white fat adipogenesis in pigs *in vivo*. Furthermore, the regulatory pathway mechanisms by which *DUSP6* affects white adipocyte proliferation and differentiation requires more studies.

## CONCLUSION

In conclusion, this is the first report on the regulatory role of *DUSP6* gene in regulating the proliferation and differentiation of porcine subcutaneous preadipocytes. Together, our data demonstrate that *DUSP6* is required for white adipocyte adipogenesis in pigs, thus warranting its consideration as an important regulator of porcine fat adipogenesis. Further exploration of the detailed mechanisms of *DUSP6* in porcine adipogenesis should lead to the identification of additional targets for the selection of fat deposition traits in pigs and the prevention and treatment of obesity.

## Figures and Tables

**Table 1:** The sequence information of RNA oligo for interference of porcine DUSP6

Name	Upstream primer sequence	Downstream primer sequence
Si-*DUSP6*-1282	GGCCAUUUCUUUCAUAGAUTT	AUCUAUGAAAGAAAUGGCCTT
Si-*DUSP6*-1402	GUCGAUGAAUGAUGCUUAUTT	AUAAGCAUCAUUCAUCGACTT
Si-*DUSP6*-857	GAAGGUGGUUUCAGUAAGUTT	ACUUACUGAAACCACCUUCTT
Negative control	UUCUCCGAACGUGUCACGUTT	ACGUGACACGUUCGGAGAATT

**Table 2:** Primers information

Gene (GeneBank number)	Primer sequence (5′–3′)	Length (bp)	Annealing temperature (°C)
*GAPDH* (NM_001206359)	F: TGGAAAGGCCATCACCATCT R: ATGGTCGTGAAGACACCAGT	105	60
*DUSP6* (NM_001267842)	F: AGTCTGACCTTGACCGAGAC R: CCCAAGTAGAGGAAGGGCAA	109	60
*PPARγ* (NM_214379)	F: TGGCCATTCGCATCTTTCAG R: ATCTCGTGGACGCCATACTT	145	60
*C/EBPβ* (NM_001199889)	F: CAGCCTTCGGAACAGTCAAG R: ATATGCAGCCGCTATGTCCT	198	60
*FASN* (NM_001099930)	F: ACTCCATCCCAGGAAAGTGG R: GAAGCAGCAGAACAGAGGTG	132	60
*FABP4* (NM_001002817)	F: CCTTCAAATTGGGCCAGGAA R: GGTGGTTGTCTTTCCATCCC	122	60
*PCNA* (NM_001291925)	F: CTGAAGAAGGTGCTGGAAGC R: TGAGACGAGTCCATGCTCTG	98	60
*CDK1* (NM_001159304)	F: TGGCCAGAAGTGGAGTCTTT R: CAGAAATTCGCTTGGCAGGA	151	60
*CDK2* (NM_001285465)	F: TACACCCATGAGGTGGTGAC R: GTCCCGAGAGTCCGAAAGAT	188	60
